# The Effect of the Nordic Hamstring Exercise on Hamstring Muscle Activity Distribution During High-Speed Running Estimated Using Multichannel Electromyography: A Pragmatic Randomized Controlled Trial

**DOI:** 10.1097/JSM.0000000000001291

**Published:** 2024-11-08

**Authors:** Jozef J. M. Suskens, Huub Maas, Jaap H. van Dieën, Gino M. M. J. Kerkhoffs, Johannes L. Tol, Gustaaf Reurink

**Affiliations:** * Department of Orthopedic Surgery and Sports Medicine, Amsterdam UMC Location University of Amsterdam, Amsterdam, the Netherlands;; † Amsterdam Movement Sciences, Sports, Amsterdam, the Netherlands;; ‡ Amsterdam Collaboration on Health & Safety in Sports (ACHSS), AMC/VUmc IOC Research Center, Amsterdam, Netherlands;; § Department of Human Movement Sciences, Faculty of Behavioural and Movement Sciences, Vrije Universiteit, Amsterdam Movement Sciences (AMS), Amsterdam, the Netherlands; and; ¶ Aspetar Orthopaedic and Sports Medicine Hospital, Doha, Qatar.

**Keywords:** hamstring injury, Nordic hamstring exercise, electromyography, high-speed running

## Abstract

**Objective:**

To evaluate the effect of the Nordic hamstring exercise on normalized muscle activity and relative contribution of the biceps femoris long head, semitendinosus, and semimembranosus through multichannel electromyography in the late-swing phase of high-speed running.

**Design:**

A pragmatic, 2-arm, single-center randomized controlled trial. Participants were randomly assigned to a Nordic group or control group.

**Setting:**

Dutch male basketball.

**Participants:**

Twenty injury-free players (mean age 18 ± 3 years).

**Intervention:**

A 12-week Nordic hamstring exercise intervention.

**Main Outcome Measures:**

Level of normalized muscle activity (percentage maximal voluntary isometric contraction [%MVIC]) and relative contribution (%con) of hamstring muscles for 12 weeks.

**Results:**

The Nordic hamstring exercise intervention did not result in significant changes for 12 weeks. For normalized muscle activity, between-group differences (compared with the control group) for 12 weeks were 11.4 %MVIC (95% confidence interval [95% CI]: −11.0, 33.8) for the biceps femoris long head, −9.4 %MVIC (95% CI: −23.3, 5.2) for the semitendinosus, and −2.7 %MVIC (95% CI: −15.8, 10.3) for the semimembranosus, *P* = 0.151. For relative contribution, between-group differences for 12 weeks were −6.1 %con (95% CI: −2.4, 14.6) for the biceps femoris long head, −7.0 %con (95% CI: −13.6, −0.4) for the semitendinosus, and 0.9 %con (95% CI: −9.2, 11.0) for the semimembranosus *P* = 0.187. Positive values are in favor of the Nordic group.

**Conclusions:**

A 12-week Nordic hamstring exercise intervention did not affect the level of muscle activity and relative contribution of hamstring muscles in the late-swing phase of high-speed running. Because of the low amount of data sets, results should be interpreted cautiously.

## Abstract 2

Supplemental Digital Content is Available in the Text.

## INTRODUCTION

Acute hamstring injuries are highly prevalent, especially in sports that involve high-speed running.^1–4^ Hamstring muscles absorb high levels of mechanical load simultaneously with high velocity muscle lengthening in the late-swing phase of a stride cycle.^5–8^ This phase is, therefore, considered as most injurious.^9^ In 9 out of 10 acute hamstring injuries, the biceps femoris long head is affected during high-speed running.^10,11^ The Nordic hamstring exercise decreases the rate of acute hamstring injuries significantly.^12,13^

Two preventive mechanisms of the Nordic hamstring exercise are mainly discussed in the literature: increased fascicle length and increased eccentric strength.^14,15^ A significant increase in fascicle length of the biceps femoris long head and semitendinosus in a passive state has been reported as a consequence of a Nordic hamstring exercise intervention.^16–22^ The risk of sarcomere overstretching might be reduced when the number of sarcomeres in series is increased, yet this presumption as an effect is argued.^23–25^ Increased eccentric strength capacity probably reduces the load on the muscle relative to failure levels. Although both effects may be related, the preventive mechanism of a Nordic hamstring exercise is yet not fully unraveled. More factors play a role in the preventive mechanism as the level of muscle activity and distribution of muscle excitation between muscles during high-speed running.^26^

Hamstring muscles are in general most active in the late-swing phase of the stride cycle during running.^27–29^ The level of muscle activity of the biceps femoris long head is in this phase comparable with the semitendinosus and semimembranosus. However, the level of muscle activity of the semimembranosus is higher than that of the semitendinosus in the same phase.^30^ Muscle activity of individual hamstring muscles differs between high-speed running and various exercises, and is thus task depending.^29–33^ It is also suggested that an imbalanced activity distribution increases the risk for a hamstring injury. The proportion of muscle activity to the summated amount of hamstring activity can be measured by electromyography (EMG) to investigate this balance. It expresses the relative contribution per individual hamstring muscle.^26,34^ A higher relative contribution of the biceps femoris long head during a strenuous eccentric exercise was predictive of a first-time injury in individual athletes.^35^ Knowing the effect of a Nordic hamstring exercise intervention on the activity distribution and relative contribution between hamstring muscles during high-speed running is, therefore, essential. An increased contribution of the semitendinosus head during high-speed running after a 12-week Nordic hamstring exercise would suggest a preventive mechanism.

Electromyography has shown to be a reliable method for measuring hamstring muscle activity during high-speed running. Electromyography outcome parameter defining amplitude (eg, integrated EMG and the root mean square) in the upper leg muscles shows good-to-excellent reliability between the 2 testing occasions.^36^

The aim of this study was to evaluate the effect of a 12-week Nordic hamstring exercise intervention on biceps femoris long head, semitendinosus, and semimembranosus muscle's activity and relative contributions through multichannel electromyography during high-speed running, primarily in the late-swing phase.

## MATERIALS AND METHODS

### Study Design and Participants

This pragmatic 2-arm, single-center, randomized controlled trial (RCT) was a substudy of a 3-arm RCT in which the efficacy of 2 hamstring injury prevention exercises on muscle fascicle length and orientation is evaluated in male basketball players (the Netherlands Trial Register ID: NL7248). This 3-arm RCT consisted of 24 participants per arm: Nordic group, Diver group, and control group.^22^ Core methods and parts of the baseline measurements were previously described.^30^ Male basketball players who were part of a basketball club as an athlete, and 16 years of age and older were included. A hamstring injury in the preceding 12 months before baseline measurement was an exclusion criterion. All participants gave written consent before the start of data collection. Study protocol and testing procedure were approved by the medical research ethics committee of the Academic Medical Center Amsterdam (NL63496.018.17) and were in accordance with the Declaration of Helsinki. Participants of the same team were randomized by cluster randomization, and individual participating participants were randomized separately. There were 2 required measurement sessions for inclusion in data analysis: a baseline measurement in the week preceding the start of the intervention and a follow-up measurement within 7 days after the 12-week intervention period.

### Intervention and Compliance

Intervention protocol for the Nordic group was based on the existing evidence-based Nordic hamstring exercise intervention protocol of *Petersen et al* and *Van der Horst et al* for 12 weeks.^12,13^ Details, such as number and frequency of sessions, are reported in Table 1. Participants in the Nordic group received details on the protocol, including a step-by-step description and video link of the exercise by mail.^37^ They were advised to execute the exercise at the end of a training, yet free to choose appropriate moments and without supervision. Participants allocated to the control group were instructed to continue their usual training regime. Both groups were asked to postpone the start of additional hamstring strengthening exercise to after the follow-up measurement session. Criteria for dichotomization of intervention compliance through online questionnaires included completion of at least 50% of the prescribed sessions. An overview of the online compliance questionnaire (CastorEDC, CIWIT B.V.) and dichotomization can be found in **Supplemental Digital Content 1**, http://links.lww.com/JSM/A462.

**TABLE 1.** Nordic Hamstring Exercise Intervention

Week Number	Sessions per Week (Frequency)	No. of Sets per Session	Repetitions per Set
1	1	2	5
2	2	2	6
3	2	3	6
4	2	3	6, 7, 8
5	2	3	8, 9, 10
6–12	2	3	10, 9, 8

### Study Preparation

#### Multichannel Electromyography

Based on previous findings of heterogeneous muscle activity in different exercises and running,^28,29^ multichannel EMG was used to minimize under- or overestimation in a single-channel EMG setup. Methods for preparation of multichannel EMG of the hamstring muscles of the left leg were described previously.^30^ In summary, electrode pairs (22 × 28 mm, Blue Sensor N-00-S, Ambu Medicotest A/S) were uniformly distributed along the proximal–distal axis of each muscle belly: 5 over the biceps femoris long head, 4 over the semitendinosus, and 6 over the semimembranosus.^30^ Signals were differentially amplified and stored on a computer through a wired connection (Porti7-16bt, TMS International BV; input impedance > 10^12^ Ω, analog–digital conversion at 2000 samples per second, 22-bit resolution).

#### Maximal Voluntary Isometric Contraction

Three maximum voluntary isometric contractions (MVIC) were measured at both measurement sessions for EMG normalization purposes. In prone position, 15 degrees knee flexion (0° anatomical zero) with the ankle in neutral position and the foot manually fixated by the examiner.^38,39^ The participants gradually increased knee flexion effort from rest to maximum and sustained the maximum for approximately 3 seconds with 30 seconds of rest in between.

#### Motion Tracking

Methods for preparation of motion tracking to measure hip and knee flexion–extension angles of the left leg were described previously.^30^ In summary, 3 dimensional coordinates of the pelvis, upper leg, lower leg, and foot were collected with 3 motion capture cameras (Optotrak Certus, Northern Digital, Waterloo, Canada), surrounding a treadmill. Sample frequency of the motion capture system was 100 Hz and data were synchronized with EMG recordings by a pulse, sent by the motion capture system and received as extra input channel in the EMG system.

### Data Collection

High-speed running trials were performed on an approximately 3 m wide by 4 m long treadmill (Bonte Technology B.V., Zwolle, the Netherlands) while wearing a safety harness. This measurement set-up was used because of the controllable treadmill speed and security measures. The participants were instructed to run in the center of the treadmill. A 5-minute warm-up of jogging at a self-selected speed started with a gentle acceleration (0.88 m·s^−2^) of the treadmill for familiarization. Muscle activity and kinematic data were collected during 3 experimental trials. During each trial, the treadmill speed accelerated up to a maximal running speed that participants subjectively could maintain for maximally 10 seconds. Participants were instructed to verbally indicate when maximal running speed was reached, after which the acceleration was manually terminated by the examiner. After approximately 3 seconds of constant speed, the treadmill decelerated because participants had to maintain running during the deceleration phase. Maximal treadmill speed was stored and used as a target speed at the follow-up measurement session. Participants were given approximately 5 minutes of self-selected resting time to recover between trials.

### Data Analysis

#### Kinematic Data

The baseline and follow-up trials with matching running speed and performed adequately within the field of view of the motion capture system were analyzed, using MATLAB R2020b (The MathWorks, Natick, MA). Methods for analyzing kinematic and electromyographic data were described previously.^30^ A stride cycle was defined from toe-off of the left foot, to the next toe-off of the same foot.^9^ Joint angles were calculated, using Euler decomposition in the order Y-X-Z (sagittal plane flexion–coronal plane flexion–transverse plane rotation) and Y–X–Z (sagittal plane flexion–coronal plane flexion–transverse plane rotation) for the hip and knee joint, respectively.^40^ Three phases were determined per stride cycle: early-swing; from toe-off to maximum knee flexion, late-swing; from maximum knee flexion to heel-strike, stance; from heel-strike to toe-off. Of 3 consecutive strides, the 3 individual phases were extracted from the multichannel EMG and averaged for 3 strides, over the corresponding electrode locations per muscle. Motion capture data of both feet for 1 high-speed running trial are illustrated in **Supplemental Digital Content 2**, http://links.lww.com/JSM/A463. Periods with evident visual contamination in the raw EMG data (eg, movement artifacts) were manually excluded.

### Primary Outcome Measures

The primary outcome measures were normalized muscle activity (percentage of maximal voluntary isometric contraction, %MVIC) and relative contribution (relative contribution (%con), ratio of the individual normalized activity of the biceps femoris long head, the semitendinosus, and the semimembranosus, to the summated normalized muscle activity of the 3 muscles), between muscles (biceps femoris long head, semitendinosus, and the semimembranosus) in the late-swing phase during high-speed running.^41,42^

### Secondary Outcome Measures

The secondary outcome measures were normalized muscle activity (%MVIC) and relative contribution (%con) between muscles (biceps femoris long head, semitendinosus, and the semimembranosus) in the early-swing and stance phase during high-speed running. Other secondary outcome measures were location of peak EMG activity in the stride cycle (in percentage stride cycle), and associated hip and knee joint angles (in degrees) at peak EMG activity per individual hamstring muscle. Neutral standing position was 0°, with positive values for flexion of the hip and knee joints. Electromyography data were time normalized for 1 complete stride (100 samples, 1%–100%), with the relative duration of the early-swing, late-swing, and stance phase set at a fixed percentage per phase, determined as the ratio of the group average duration of the individual phases with respect to the total stride duration.

### Sample Size

Sample size calculation in the 3-arm RCT yielded 24 participants per group.^22^ For this specific substudy, we did not a priori calculate the sample size.

### Statistical Analysis

Descriptive statistics (means and standard deviations) were used to describe baseline characteristics, including age, body height, and mass. Differences between groups at baseline were tested with independent samples *t*-tests. For the analysis of the outcome measures muscle activity and relative contribution per phase (both primary and secondary outcome measures), a general linear model for repeated measures was used (repeated within-subjects factors); muscle (biceps femoris long head, semitendinosus, semimembranosus), nonrepeated between-subjects factor; group (Nordic group, control group). In a per-protocol sensitivity analysis of the primary outcome measures, all participants of the control group and only the compliant participants of the Nordic group were included. For the analysis of the secondary outcome measures instant of peak EMG, hip and knee joint angle at peak EMG, a general linear model for repeated measures was used to test for the effect of a Nordic hamstring exercise intervention on the between-group differences for 12 weeks: repeated within-subjects factors; muscle (biceps femoris long head, semitendinosus, semimembranosus), nonrepeated between-subjects factor; group (Nordic group, control group). Absolute changes from baseline to follow-up were included in all tests to test for the effect of the intervention on the between-group differences for 12 weeks. For all repeated measures tests, when sphericity was violated (Mauchly test *P* < 0.05), the Greenhouse–Geisser correction was used. The level of significance was set at an alpha of 0.05. In the event of a statistically significant interaction effect, predefined *post hoc* analyses were performed using Bonferroni correction. All positive between-group values were in favor of the Nordic group. Statistical analyses were performed using IBM SPSS Statistics (IBM SPSS Statistics for Windows, Version 27.0).

## RESULTS

### Data Collection

**Figure 1.**
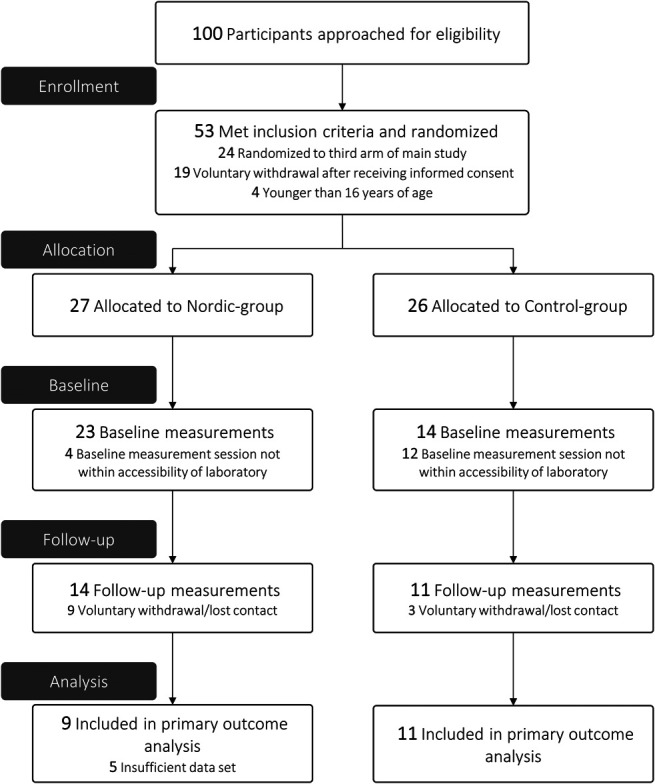
Flow diagram of participants through the study.

Between September 2018 and March 2020, 100 basketball players were approached to participate in the 3-arm RCT, of whom 53 participants showed interest and met the inclusion criteria. Participants were randomized to a Nordic group (n = 27) or control group (n = 26) in this 2-arm RCT. Sixteen included participants from the main 3-arm RCT could not participate in this study because their baseline measurement was outside the period that the laboratory was accessible (Nordic group, n = 4; control group, n = 12). Twenty-five participants (Nordic group, n = 14; control group, n = 11) completed a measurement session at baseline and follow-up in this study (Figure 1). Participants were measured throughout the entire season, disregarding in-season and off-season training schedules. In 5 data sets of the Nordic group, we encountered a technical issue and data were not useable for analysis. Seven episodes of evident contamination were observed in the raw EMG data sets of 7 individual participants in either the baseline or follow-up measurement. Eventually, 20 participants were used for statistical analysis (Nordic group, n = 9; control group, n = 11). Based on the group averaged absolute durations, a stride cycle (1%–100%) was divided in the relative durations: 1% to 42% stride cycle for the early-swing phase, 43% to 80% stride cycle for the late-swing phase, and 81% to 100% stride cycle for the stance phase. Baseline characteristics are presented in Table 2.

**TABLE 2.** Baseline Characteristics of Participants in the Nordic Group and Control Group

Participant Characteristics	Nordic Group (n = 9)	Control Group (n = 11)	Between-Group Mean Difference (95% CI)
Age (yr)	17.7 ± 0.9	19.0 ± 3.6	1.3 (−1.1 to 3.8)
Body height (cm)	193.1 ± 7.9	192.0 ± 10.2	−1.1 (−9.6 to 7.4)
Mass (kg)	82.4 ± 7.9	85.5 ± 12.2	3.0 (−6.5 to 12.5)
Running speed (km.h^−1^)	27.3 ± 1.0	27.8 ± 1.7	0.5 (−0.9 to 1.8)

Activity Distribution Between Muscles		Nordic Group	Control Group	Between-Group Mean Difference (95% CI)
Normalized muscle activity (%MVIC)	Biceps femoris	43.8 ± 20.5	63.2 ± 33.0	19.3 (−7.4 to 46.1)
	Semitendinosus	36.8 ± 10.0	39.0 ± 10.6	2.2 (−7.6 to 11.9)
	Semimembranosus	40.1 ± 22.4	40.4 ± 11.9	0.2 (−16.2 to 16.7)
Relative contribution (%con)	Biceps femoris	37.9 ± 7.1	43.1 ± 13.1	5.3 (−5.0 to 15.5)
	Semitendinosus	30.6 ± 4.2	27.0 ± 4.5	−3.6 (−7.7 to 0.5)
	Semimembranosus	31.5 ± 8.5	29.8 ± 10.9	−1.7 (−11.0 to 7.6)

Muscle activity was measured through multichannel electromyography in the late-swing phase of high-speed running.

Values are means ± standard deviations.

%con, percentage relative contribution; CI, confidence interval; %MVIC, percentage maximal voluntary contraction.

### Primary Outcome Measures

**Figure 2.**
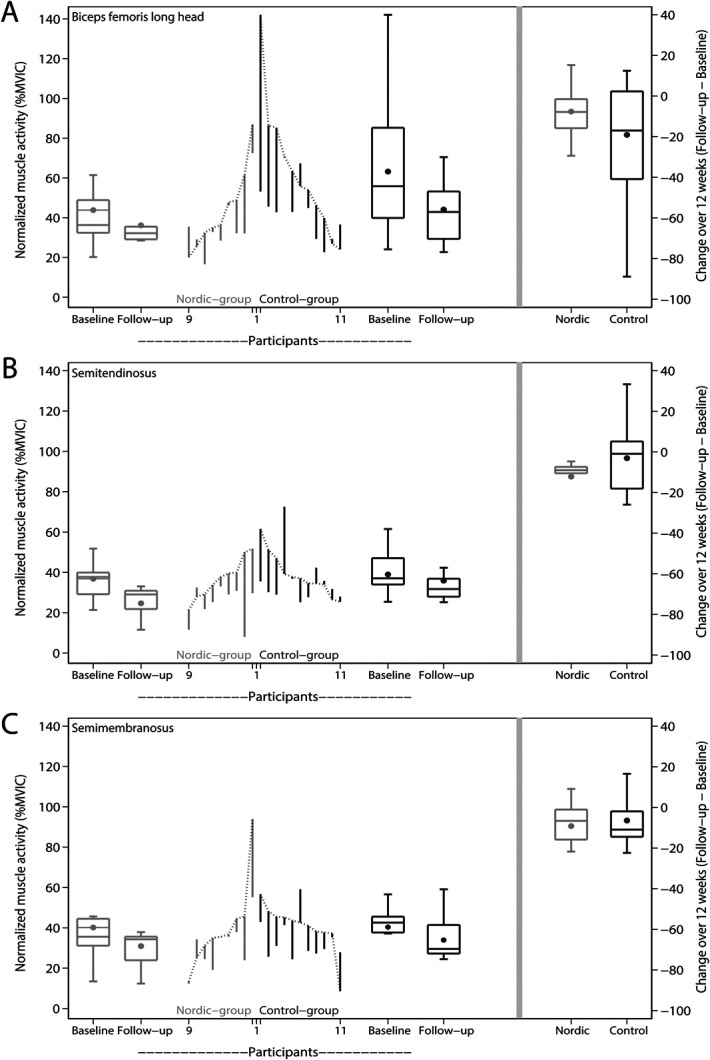
Change in normalized muscle activity for each participant of the Nordic group and control group for 12 weeks. Muscle activity was measured through multichannel electromyography in the late-swing phase during high-speed running. A, Biceps femoris long head. B, Semitendinosus. C, Semimembranosus. Changes from baseline to 12 weeks are represented by the vertical lines. Upward and downward lines indicate increases or decreases, respectively. The horizontal lines in the boxplots from bottom to top represent the 25th, 50th (median), and 75th percentile. The dots within the boxplot represent the mean. The whiskers show the lowest and highest value.

The Nordic hamstring exercise intervention did not result in significant changes in normalized muscle activity of any of the 3 hamstring muscles for 12 weeks (nonsignificant effect *Muscle x Intervention*, *F*(1.5, 27.1) = 2.1, *P* = 0.151). Between-group differences for 12 weeks were 11.4 %MVIC (95% confidence interval [95% CI]: −11.0, 33.8) for the biceps femoris long head, −9.4 %MVIC (95% CI: −23.3, 5.2) for the semitendinosus and −2.7 %MVIC (95% CI: −15.8, 10.3) for the semimembranosus (Table 3 and Figure 2).^43^ Positive values are in favor of the Nordic group.

**TABLE 3.** Primary and Secondary Outcomes at Follow-Up and For 12 Weeks

		Nordic Group (n = 9)		Control Group (n = 11)		Between-Group Difference (95% CI)	*P*
		Follow-Up	Absolute Δ Over 12 wk	Follow-Up	Absolute Δ for 12 wk		
Primary outcome measures							
Normalized muscle activity (%MVIC)	Biceps femoris	36.2 ± 15.6	−7.7 ± 13.1	44.1 ± 15.1	−19.1 ± 29.6	11.4 (−11.0 to 33.8)	0.151
	Semitendinosus	24.7 ± 9.2	−12.2 ± 13.1	35.9 ± 13.1	−3.1 ± 16.5	−9.4 (-23.3 to 5.2)	
	Semimembranosus	30.9 ± 12.6	−9.2 ± 14.3	33.9 ± 10.3	−6.5 ± 13.4	−2.7 (-15.8 to 10.3)	
Relative contribution (%con)	Biceps femoris	39.5 ± 7.2	1.6 ± 8.5	38.7 ± 7.9	−4.5 ± 9.4	6.1 (-2.4 to 14.6)	0.187
	Semitendinosus	27.7 ± 9.0	−3.0 ± 6.8	31.1 ± 6.1	4.0 ± 7.1	−7.0 (-13.6 to 0.4)	
	Semimembranosus	32.9 ± 7.5	1.4 ± 9.8	30.3 ± 5.6	0.4 ± 11.4	0.9 (-9.2 t o11.0)	
Secondary outcome measures							
Instant of peak EMG activity (% stride cycle)	Biceps femoris	73.3 ± 14.2	4.4 ± 23.2	66.4 ± 13.5	6.8 ± 21.1	−2.4 (-23.2 to 18.5)	0.075
	Semitendinosus	76.9 ± 10.6	7.2 ± 10.2	64.8 ± 13.9	−8.1 ± 16.9	15.3 (1.8 to 28.8)	
	Semimembranosus	65.0 ± 5.5	−7.7 ± 11.6	66.8 ± 5.0	−2.4 ± 9.4	−5.3 (-15.2 to 4.6)	
Hip joint angle at peak EMG activity (°)	Biceps femoris	51.3 ± 22.7	−6.1 ± 37.0	48.6 ± 19.6	−12.0 ± 25.0	5.8 (−23.3 to 35.0)	0.386
	Semitendinosus	47.8 ± 20.6	−16.5 ± 20.1	49.7 ± 20.6	−6.6 ± 30.7	−9.9 (−34.9 to 15.1)	
	Semimembranosus	66.5 ± 7.9	5.6 ± 19.8	55.8 ± 10.8	−4.4 ± 18.1	10.0 (−7.8 to 27.9)	
Knee joint angle at peak EMG activity (°)	Biceps femoris	49.9 ± 27.0	−12.2 ± 46.1	52.4 ± 28.1	−22.8 ± 58.1	10.3 (−39.8 to 60.5)	0.128
	Semitendinosus	37.5 ± 10.0	−15.1 ± 21.1	56.3 ± 26.3	14.6 ± 30.0	−29.7 (−54.7 to −4.7)	
	Semimembranosus	53.3 ± 20.4	2.6 ± 17.2	46.5 ± 20.3	−6.1 ± 24.7	8.7 (−11.8 to 29.2)	

Muscle activity was measured through multichannel electromyography in the late-swing phase of high-speed running.

Values are means ± standard deviations.

%MVIC, percentage maximal voluntary contraction; %con, percentage relative contribution; Δ, follow-up minus baseline; CI, confidence interval; *P,* probability value interaction effect.

**Figure 3.**
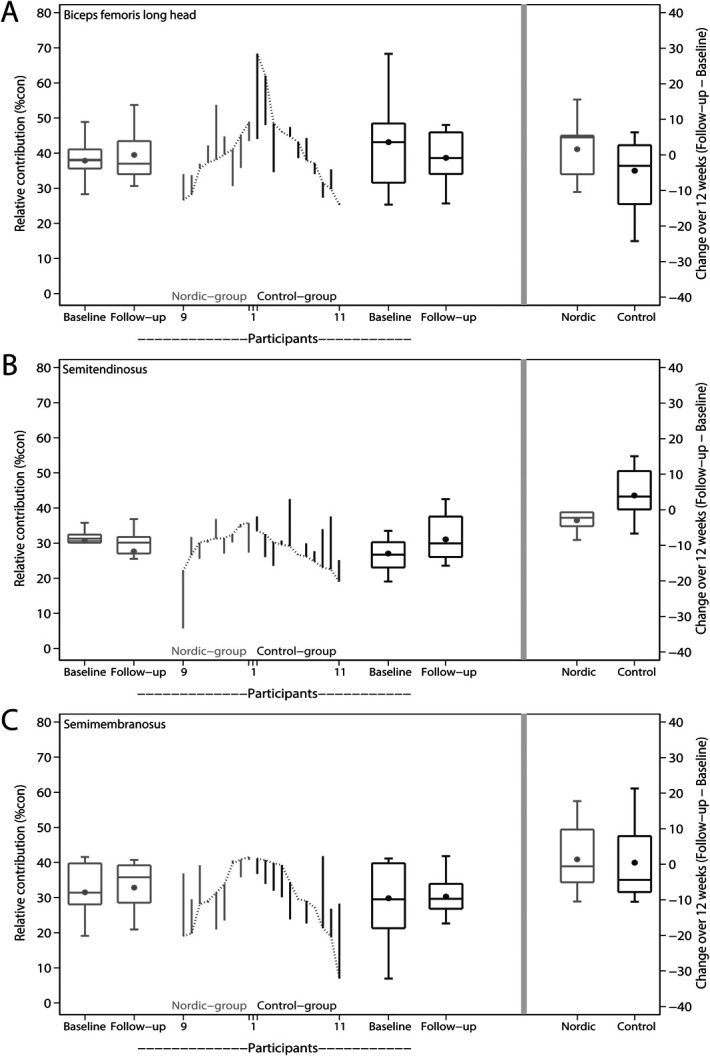
Change in relative contribution for each participant of the Nordic group and control group for 12 weeks. Relative contribution was measured through multichannel electromyography in the late-swing phase during high-speed running. A, Biceps femoris long head. B, Semitendinosus. C, Semimembranosus. Changes from baseline to 12 weeks are represented by the vertical lines. Upward and downward lines indicate increases or decreases, respectively. The horizontal lines in the boxplots from bottom to top represent the 25th, 50th (median), and 75th percentile. The dots within the boxplot represent the mean. The whiskers show the lowest and highest value.

The Nordic hamstring exercise intervention did not result in significant changes in relative contribution of any of the 3 hamstring muscles for 12 weeks (nonsignificant effect *Muscle x Intervention*, *F*(2, 36) = 1.8, *P* = 0.187). Between-group differences for 12 weeks were 6.1 %con (95% CI: −2.4, 14.6) for the biceps femoris long head, −7.0 %con (95% CI: −13.6, −0.4) for the semitendinosus, and 0.9 %con (95% CI: −9.2, 11.0) for the semimembranosus (Table 3 and Figure 3). Five participants (56%) were considered to be compliant to the exercise protocol and used for the per-protocol analysis. Results of the per-protocol analysis are described in **Supplemental Digital Content 3**, http://links.lww.com/JSM/A464.

### Secondary Outcome Measures

In the early-swing phase, the Nordic hamstring exercise intervention did not result in significant changes in normalized muscle activity (*F*(2, 36) = 0.3, *P* = 0.768) or relative contribution (*F*(2, 36) = 0.0, *P* = 0.969) of any of the 3 hamstring muscles for 12 weeks. In the stance phase, the Nordic hamstring exercise intervention did not result in significant changes in normalized muscle activity (*F*(1.5, 27.4 = 0.5, *P* = 0.580) or relative contribution (*F*(2, 36) = 0.5, *P* = 0.597) of any of the 3 hamstring muscles for 12 weeks. Detailed results and illustrations of these 2 phases are described in **Supplemental Digital Content 4**, http://links.lww.com/JSM/A465.

The Nordic hamstring exercise intervention did not result in significant changes in instant of peak EMG activity of any of the 3 hamstring muscles for 12 weeks (nonsignificant interaction effect *Muscle x Intervention*, *F*(2, 36) = 2.8, *P* = 0.075). Between-group differences for 12 weeks were −2.4% stride cycle (95% CI: −23.2, 18.5) for the biceps femoris long head, 15.3% stride cycle (95% CI: 1.8, 28.8) for the semitendinosus, and −5.3% stride cycle (95% CI: −15.2, 4.6) for the semimembranosus.

The Nordic hamstring exercise intervention did not result in significant changes in hip joint angle at instant of peak EMG of any of the 3 hamstring muscles for 12 weeks (nonsignificant interaction effect *Muscle* × *Intervention*, *F*(2, 36) = 1.0, *P* = 0.386). Between-group differences for 12 weeks were 5.8 degrees (95% CI: −23.3, 35.0) for the biceps femoris long head, −9.9 degrees (95% CI: −34.9, 15.1) for the semitendinosus, and 10.0° (95% CI: −7.8, 27.9) for the semimembranosus.

The Nordic hamstring exercise intervention did not result in significant changes in knee joint angle at instant of peak EMG of any of the 3 hamstring muscles for 12 weeks (nonsignificant interaction effect *Muscle* × *Intervention*, *F*(1.4, 25.1) = 2.4, *P* = 0.128). Between-group differences for 12 weeks were 10.3 degrees (95% CI: −39.8, 60.5) for the biceps femoris long head, −29.7 degrees (95% CI: −54.7, −4.7) for the semitendinosus, and 8.7 degrees (95% CI: −11.8, 29.2) for the semimembranosus.

## DISCUSSION

In this 2-arm RCT among basketball players, a 12-week Nordic hamstring exercise intervention, compared with usual training, did not significantly affect normalized muscle activity or relative contribution of the hamstring muscles (ie, biceps femoris long head, semitendinosus, and semimembranosus) during high-speed running. Also instant of peak EMG activity during high-speed running and the corresponding hip and knee joint angles were not significantly affected by a Nordic hamstring exercise intervention. The pragmatic RCT design and missing data possibly led to underpowered analysis.

This is the first RCT evaluating the effects of Nordic hamstring exercises on the hamstring muscles' individual muscle activity and relative contribution during high-speed running. A review with meta-analysis on the effects of Nordic hamstring exercises showed beneficial effects on sprint performance.^15^ For the 5, 10, and 20 m sprint, performance improved on average 2.2%.^15^ Maximal sprint velocity increase with 1.4% after a 4-week Nordic hamstring exercise intervention.^44^ Eccentric strength capacity increased in that study, which might be associated with improved sprint capacity. This cannot be confirmed by our results, as no strength measurements were performed in this study.

Previous studies reported significantly higher muscle activity of the semitendinosus compared with the biceps femoris long head during a Nordic hamstring exercise,^32,45^ suggesting a superior training stimuli.^46^ It did not cause a change in muscle activity of the semitendinosus during high-speed running in our population. We previously reported no significant effect of a Nordic hamstring exercise intervention on hamstring muscle activity and relative contributions during the performance of a Nordic hamstring exercise.^47^ EMG is reported to be a reliable method for describing hamstring muscle activity during high-speed running.^36^ The variety in equipment and processing methods limits data comparison between studies. This study serves predominantly as a methodological example of how muscle activity assesses the possible effect of an exercise intervention over time. There is a general consensus that EMG normalization is essential for comparison within participants over time and between participants when comparing EMG muscle activity amplitudes. However, there is no consensus on the preferable method for normalizing EMG data.^48^ Maximal voluntary isometric contractions were used in this study. This type of contraction might be insensitive to the effects on a Nordic hamstring exercise intervention, as there is also an absence of an effect on isometric hamstring strength.

One of the strengths of this study was the use of multichannel EMG. Individual hamstring muscles have a heterogeneous muscle activity distribution during all phases of high-speed running, which makes single channel EMG prone to over- or underestimation of muscle activity.^28,29^ In addition, running speed was standardized between measurement sessions.

The main limitation of this study was the low number of data sets available for analysis and the risk of a type II error. Participant recruitment was less successful than expected within the limited time frame with accessibility of the laboratory. In 5 data sets, we encountered a technical issue, which made them unusable for analysis. No post hoc power analyses were computed, because in relatively small study populations these estimations can be misleading and too variable to be informative for the true effect size.^49^ Second, we used a subjective definition of “high-speed” running based on self-selection on a treadmill. We did not assess baseline maximal running speed outside the laboratory. There is no clear absolute or relative cutoff for running speed in the literature described. Third, the individual hamstring muscles were manually identified by palpation. Despite clinical expertise and comparison with magnetic resonance imaging obtained in the 3-arm RCT, ultrasound might have been an additional tool to more objectively define muscle boundaries and select electrode locations. Fourth, some degree of cross-talk of EMG signals from adjacent muscles cannot be ruled out, yet we averaged muscle activity for multiple electrode locations per muscle.

## CONCLUSIONS

In conclusion, this pragmatic 2-arm study showed no effect of a Nordic hamstring exercise intervention on normalized muscle activity and relative contribution of hamstring muscles during high-speed running among injury-free basketball players. Given the risk of a type II error, the results should be cautiously interpreted.

## Supplementary Material
