# Supplementary material for: The Effect of the Nordic Hamstring Exercise on Hamstring Muscle Activity Distribution During High-Speed Running Estimated Using Multichannel Electromyography: A Pragmatic Randomized Controlled Trial
Source: Clin J Sport Med. 2024 Nov 8;35(2):103–12. doi: 10.1097/JSM.0000000000001291 (PMC11837967; doi:10.1097/JSM.0000000000001291)
Supplement: Supplementary file 1 [file cjsm-35-103-s001.docx]

# SUPPLEMENTAL DIGITAL CONTENT 1

# Title

The effect of the Nordic hamstring exercise on hamstring muscle activity distribution during high-speed running estimated using multichannel electromyography: a pragmatic randomized controlled trial

# Author information

Jozef JM Suskens^1,2,3^, Huub Maas^2,4^, Jaap H van Dieën^2,4^, Gino MMJ Kerkhoffs^1,2,3^, Johannes L Tol^2,3,5^, Gustaaf Reurink^2,3^

# Affiliations

1. Amsterdam UMC location University of Amsterdam, Department of Orthopedic Surgery and Sports Medicine, Meibergdreef 9, Amsterdam, The Netherlands
2. Amsterdam Movement Sciences, Sports, Amsterdam, The Netherlands
3. Amsterdam Collaboration on Health & Safety in Sports (ACHSS), AMC/VUmc IOC Research Center, Amsterdam, Netherlands
4. Department of Human Movement Sciences, Faculty of Behavioural and Movement Sciences, Vrije Universiteit, Amsterdam Movement Sciences (AMS), Amsterdam, The Netherlands
5. Aspetar Orthopaedic and Sports Medicine Hospital, Doha, Qatar

# Corresponding author

Correspondence to Jozef JM Suskens; [j.j.suskens@amsterdamumc.nl](mailto:j.j.suskens@amsterdamumc.nl)

ORCID: 0000-0003-0878-3946

**Table S1.** Online questionnaires (biweekly and at the end of intervention period) to assess compliance

| When | Question |
| --- | --- |
| *Biweekly* | |
| Biweekly question 1 | In the two preceding weeks, how often did you execute the exercises according to the provided exercise protocol? This is regarding the number of sessions, not about the specific number of sets per training: *Possible answer: number 0-4.* |
| *Evaluation at the end of intervention period* | |
| Evaluation question 1 | Each week, I executed the prescribed exercises at least one time per week. *Possible answer:* *yes/no.* |
| Evaluation question 2 | If not, specify how many weeks you did execute the prescribed exercises: *Possible answer: number 0-12.* |


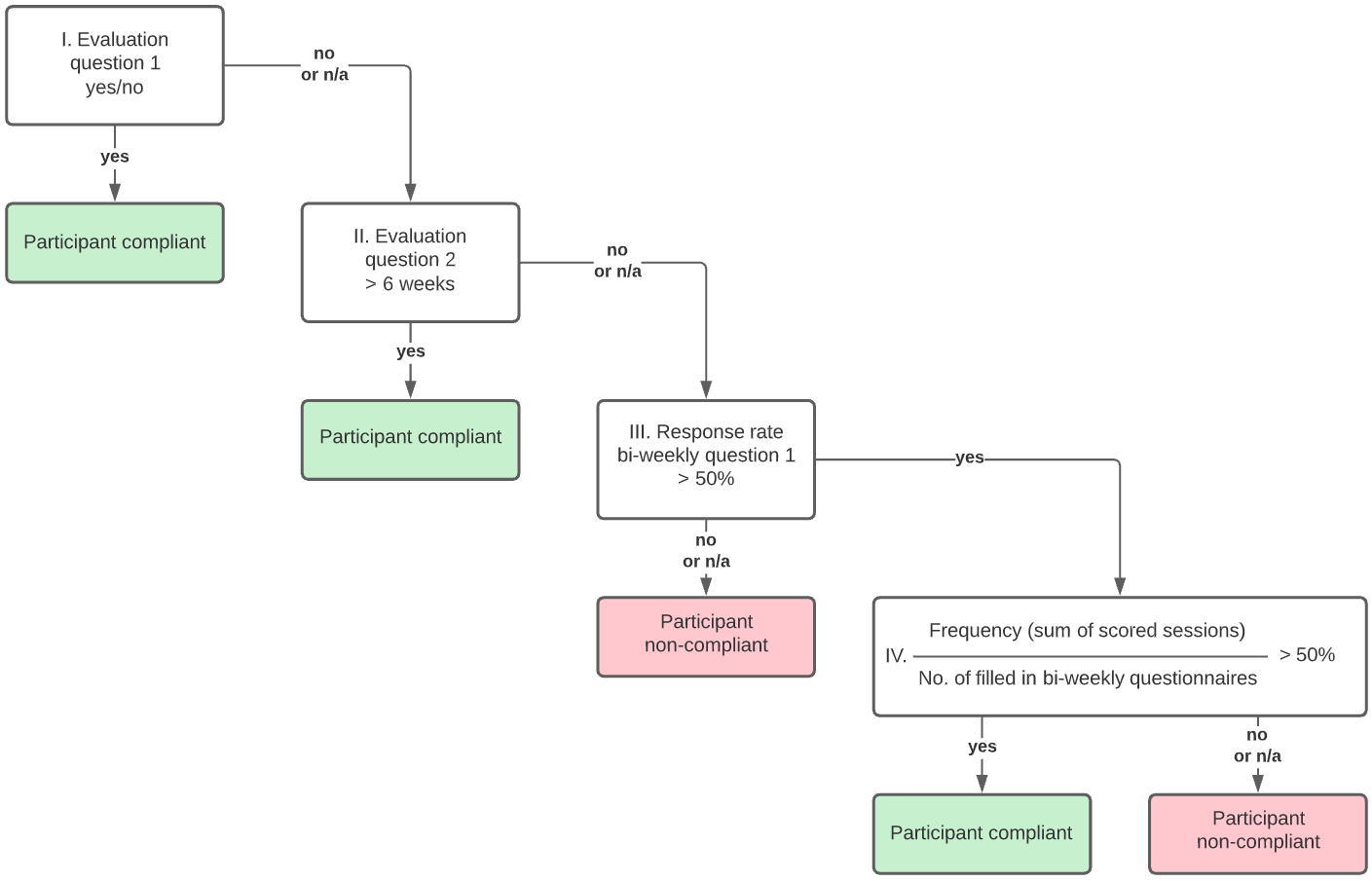


**Figure S1.** Decision tree to categorize compliance
