# Supplementary material for: The Effect of the Nordic Hamstring Exercise on Hamstring Muscle Activity Distribution During High-Speed Running Estimated Using Multichannel Electromyography: A Pragmatic Randomized Controlled Trial
Source: Clin J Sport Med. 2024 Nov 8;35(2):103–12. doi: 10.1097/JSM.0000000000001291 (PMC11837967; doi:10.1097/JSM.0000000000001291)
Supplement: Supplementary file 2 [file cjsm-35-103-s002.docx]

# SUPPLEMENTAL DIGITAL CONTENT 2

# Title

The effect of the Nordic hamstring exercise on hamstring muscle activity distribution during high-speed running estimated using multichannel electromyography: a pragmatic randomized controlled trial

# Author information

Jozef JM Suskens^1,2,3^, Huub Maas^2,4^, Jaap H van Dieën^2,4^, Gino MMJ Kerkhoffs^1,2,3^, Johannes L Tol^2,3,5^, Gustaaf Reurink^2,3^

# Affiliations

1. Amsterdam UMC location University of Amsterdam, Department of Orthopedic Surgery and Sports Medicine, Meibergdreef 9, Amsterdam, The Netherlands
2. Amsterdam Movement Sciences, Sports, Amsterdam, The Netherlands
3. Amsterdam Collaboration on Health & Safety in Sports (ACHSS), AMC/VUmc IOC Research Center, Amsterdam, Netherlands
4. Department of Human Movement Sciences, Faculty of Behavioural and Movement Sciences, Vrije Universiteit, Amsterdam Movement Sciences (AMS), Amsterdam, The Netherlands
5. Aspetar Orthopaedic and Sports Medicine Hospital, Doha, Qatar

# Corresponding author

Correspondence to Jozef JM Suskens; [j.j.suskens@amsterdamumc.nl](mailto:j.j.suskens@amsterdamumc.nl)

ORCID: 0000-0003-0878-3946


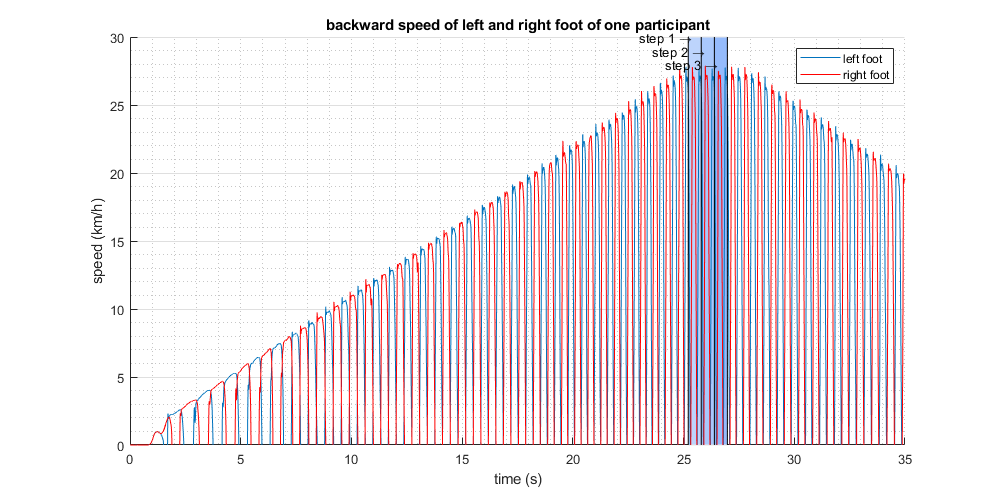


**Figure S2.** Participant G – baseline measurement**.** Backward speed of the left (blue) and right (red) foot over one high-speed running trial. Light blue shaded areas indicate the selected strides used for data analysis.
