# Supplementary material for: The Effect of the Nordic Hamstring Exercise on Hamstring Muscle Activity Distribution During High-Speed Running Estimated Using Multichannel Electromyography: A Pragmatic Randomized Controlled Trial
Source: Clin J Sport Med. 2024 Nov 8;35(2):103–12. doi: 10.1097/JSM.0000000000001291 (PMC11837967; doi:10.1097/JSM.0000000000001291)
Supplement: Supplementary file 3 [file cjsm-35-103-s003.docx]

# SUPPLEMENTAL DIGITAL CONTENT 3

# Title

The effect of the Nordic hamstring exercise on hamstring muscle activity distribution during high-speed running estimated using multichannel electromyography: a pragmatic randomized controlled trial

# Author information

Jozef JM Suskens^1,2,3^, Huub Maas^2,4^, Jaap H van Dieën^2,4^, Gino MMJ Kerkhoffs^1,2,3^, Johannes L Tol^2,3,5^, Gustaaf Reurink^2,3^

# Affiliations

1. Amsterdam UMC location University of Amsterdam, Department of Orthopedic Surgery and Sports Medicine, Meibergdreef 9, Amsterdam, The Netherlands
2. Amsterdam Movement Sciences, Sports, Amsterdam, The Netherlands
3. Amsterdam Collaboration on Health & Safety in Sports (ACHSS), AMC/VUmc IOC Research Center, Amsterdam, Netherlands
4. Department of Human Movement Sciences, Faculty of Behavioural and Movement Sciences, Vrije Universiteit, Amsterdam Movement Sciences (AMS), Amsterdam, The Netherlands
5. Aspetar Orthopaedic and Sports Medicine Hospital, Doha, Qatar

# Corresponding author

Correspondence to Jozef JM Suskens; [j.j.suskens@amsterdamumc.nl](mailto:j.j.suskens@amsterdamumc.nl)

ORCID: 0000-0003-0878-3946

Data sets of five compliant participants (56%) in the Nordic-group were used for the per-protocol group analysis. Individual results of intervention compliance are presented below (Table S4.1).

**Table S2.** Results compliance questionnaires per individual

| **Participant** | **Statement I.** | **Statement II.** | **Statement III.** | **Statement IV.** | **Compliance** |
| --- | --- | --- | --- | --- | --- |
| *A* | Yes |  |  |  | Compliant |
| *B* | No | No: n/a | Yes: 5/6 | Yes: 20/20 | Compliant |
| *C* | Yes |  |  |  | Compliant |
| *D* | No | No: n/a | No: 1/6 |  | Non-compliant |
| *E* | No | No: n/a | No: 2/6 |  | Non-compliant |
| *F* | No | No: n/a | Yes: 5/6 | Yes: 13/20 | Compliant |
| *G* | No | No: n/a | No: 1/6 |  | Non-compliant |
| *H* | No | No: n/a | No: 3/6 |  | Non-compliant |
| *I* | No | No: n/a | Yes: 6/6 | Yes: 17/24 | Compliant |

During the late-swing phase, the Nordic hamstring exercise intervention did not result in significant changes in normalized muscle activity (*F*(2,28) = 1.6, *p* = 0.219) and relative contribution (*F*(2,28) = 1.4, *p* = 0.261) of any of the three hamstring muscles. Detailed results are presented below in Table S4.3.

**Table S3.** Results per-protocol sensitivity analysis in the late-swing phase of high-speed running.

|  | | | **Nordic-group**  **(n = 5)** | | | | | | **Control-group**  **(n = 11)** | | | | | | |  | | | | | | |  | |
| --- | --- | --- | --- | --- | --- | --- | --- | --- | --- | --- | --- | --- | --- | --- | --- | --- | --- | --- | --- | --- | --- | --- | --- | --- |
|  | | | Follow-up | | | Absolute Δ over 12 weeks | | | Follow-up | | | Absolute Δ over 12 weeks | | | | Between-group difference  (95% CI) | | | | | | | *p* | |
| Normalized | | Biceps femoris | 42.5 | ± | 18.1 | -8.5 | ± | 6.9 | 44.1 | ± | 15.1 | -19.1 | ± | 29.6 | -1.7 | | ( | -20.2 | to | 16.9 | ) | *0.219* | |  |
| muscle activity | | Semitendinosus | 25.3 | ± | 10.1 | -14.9 | ± | 15.3 | 35.9 | ± | 13.1 | -3.1 | ± | 16.5 | -10.6 | | ( | -24.9 | to | 3.6 | ) |  | |  |
| (%MVIC) |  | Semimembranosus | 31.4 | ± | 14.4 | -14.8 | ± | 17.8 | 33.9 | ± | 10.3 | -6.5 | ± | 13.4 | -2.5 | | ( | -16.0 | to | 11.0 | ) |  | |  |
|  |  |  |  |  |  |  |  |  |  |  |  |  |  |  |  | |  |  |  |  |  |  | |  |
| Relative | | Biceps femoris | 42.7 | ± | 7.2 | 3.5 | ± | 8.9 | 38.7 | ± | 7.9 | -4.5 | ± | 9.4 | 4.0 | | ( | -4.9 | to | 12.9 | ) | *0.261* | |  |
| contribution | | Semitendinosus | 27.2 | ± | 12.5 | -3.0 | ± | 9.4 | 31.1 | ± | 6.1 | 4.0 | ± | 7.1 | -3.8 | | ( | -13.6 | to | 5.9 | ) |  | |  |
| (%con) |  | Semimembranosus | 30.1 | ± | 8.5 | -0.6 | ± | 11.5 | 30.3 | ± | 5.6 | 0.4 | ± | 11.4 | -0.2 | | ( | -7.8 | to | 7.4 | ) |  | |  |
|  |  |  |  |  |  |  |  |  |  |  |  |  |  |  |  | |  |  |  |  |  |  | |  |

*Values are means ± standard deviations, Δ; follow-up minus baseline, CI; confidence interval, p; probability value interaction effect, %MVIC; percentage maximal voluntary contraction, %con; percentage relative contribution.*
