# Supplementary material for: The Effect of the Nordic Hamstring Exercise on Hamstring Muscle Activity Distribution During High-Speed Running Estimated Using Multichannel Electromyography: A Pragmatic Randomized Controlled Trial
Source: Clin J Sport Med. 2024 Nov 8;35(2):103–12. doi: 10.1097/JSM.0000000000001291 (PMC11837967; doi:10.1097/JSM.0000000000001291)
Supplement: Supplementary file 4 [file cjsm-35-103-s004.docx]

# SUPPLEMENTAL DIGITAL CONTENT 4

# Title

The effect of the Nordic hamstring exercise on hamstring muscle activity distribution during high-speed running estimated using multichannel electromyography: a pragmatic randomized controlled trial

# Author information

Jozef JM Suskens^1,2,3^, Huub Maas^2,4^, Jaap H van Dieën^2,4^, Gino MMJ Kerkhoffs^1,2,3^, Johannes L Tol^2,3,5^, Gustaaf Reurink^2,3^

# Affiliations

1. Amsterdam UMC location University of Amsterdam, Department of Orthopedic Surgery and Sports Medicine, Meibergdreef 9, Amsterdam, The Netherlands
2. Amsterdam Movement Sciences, Sports, Amsterdam, The Netherlands
3. Amsterdam Collaboration on Health & Safety in Sports (ACHSS), AMC/VUmc IOC Research Center, Amsterdam, Netherlands
4. Department of Human Movement Sciences, Faculty of Behavioural and Movement Sciences, Vrije Universiteit, Amsterdam Movement Sciences (AMS), Amsterdam, The Netherlands
5. Aspetar Orthopaedic and Sports Medicine Hospital, Doha, Qatar

# Corresponding author

Correspondence to Jozef JM Suskens; [j.j.suskens@amsterdamumc.nl](mailto:j.j.suskens@amsterdamumc.nl)

ORCID: 0000-0003-0878-3946

**Table S4.** Primary outcome measures at follow-up and over 12 weeks. Muscle activity was measured through multichannel electromyography in the early-swing phase of high-speed running.

| **Early-swing phase** | | | **Nordic-group**  **(n = 9)** | | | | | | **Control-group**  **(n = 11)** | | | | | | |  | | | | | | |  | |
| --- | --- | --- | --- | --- | --- | --- | --- | --- | --- | --- | --- | --- | --- | --- | --- | --- | --- | --- | --- | --- | --- | --- | --- | --- |
|  | | | Follow-up | | | Absolute Δ over 12 weeks | | | Follow-up | | | Absolute Δ over  12 weeks | | | | Between-group  difference (95% CI) | | | | | | | *p* | |
| Normalized muscle | | Biceps femoris | 10.2 | ± | 7.3 | -2.6 | ± | 10.3 | 13.0 | ± | 6.3 | -2.7 | ± | 8.8 | 0.1 | | ( | -8.9 | to | 9.1 | ) | *0.768* | |  |
| activity (%MVIC) | | Semitendinosus | 7.0 | ± | 5.5 | -2.5 | ± | 7.4 | 11.0 | ± | 8.1 | 0.3 | ± | 9.0 | -2.7 | | ( | -10.6 | to | 5.1 | ) |  | |  |
|  |  | Semimembranosus | 6.9 | ± | 4.8 | -1.6 | ± | 6.9 | 8.2 | ± | 3.9 | -0.5 | ± | 4.5 | 1.1 | | ( | -6.5 | to | 4.3 | ) |  | |  |
|  |  |  |  |  |  |  |  |  |  |  |  |  |  |  |  | |  |  |  |  |  |  | |  |
| Relative | | Biceps femoris | 43.1 | ± | 9.5 | 0.1 | ± | 10.8 | 42.5 | ± | 8.8 | -0.7 | ± | 9.7 | 0.8 | | ( | -8.9 | to | 10.5 | ) | *0.969* | |  |
| contribution | | Semitendinosus | 29.9 | ± | 11.8 | -2.4 | ± | 11.5 | 30.6 | ± | 9.2 | -1.1 | ± | 10.7 | -1.3 | | ( | -11.8 | to | 9.1 | ) |  | |  |
| (%con) |  | Semimembranosus | 27.0 | ± | 11.1 | 2.3 | ± | 13.4 | 26.9 | ± | 8.7 | 1.8 | ± | 13.4 | 0.5 | | ( | -12.1 | to | 13.1 | ) |  | |  |
|  |  |  |  |  |  |  |  |  |  |  |  |  |  |  |  | |  |  |  |  |  |  | |  |

*Values are means ± standard deviations, Δ; follow-up minus baseline, CI; confidence interval, p; probability value interaction effect, %MVIC; percentage maximal voluntary contraction, %con; percentage relative contribution.*

**Table S5.** Primary and secondary outcomes at follow-up and over 12 weeks. Muscle activity was measured through multichannel electromyography in the stance phase of high-speed running.

| **Stance phase** | | | **Nordic-group**  **(n = 9)** | | | | | | **Control-group**  **(n = 11)** | | | | | | |  | | | | | | |  | |
| --- | --- | --- | --- | --- | --- | --- | --- | --- | --- | --- | --- | --- | --- | --- | --- | --- | --- | --- | --- | --- | --- | --- | --- | --- |
|  | | | Follow-up | | | Absolute Δ over 12 weeks | | | Follow-up | | | Absolute Δ over  12 weeks | | | | Between-group  difference (95% CI) | | | | | | | *p* | |
| Normalized muscle | | Biceps femoris | 31.4 | ± | 10.0 | -7.2 | ± | 13.7 | 29.4 | ± | 14.7 | -12.9 | ± | 27.1 | 5.7 | | ( | -15.3 | to | 26.7 | ) | *0.580* | |  |
| activity (%MVIC) | | Semitendinosus | 23.2 | ± | 10.2 | -8.9 | ± | 14.6 | 27.1 | ± | 12.7 | -7.7 | ± | 20.4 | -1.2 | | ( | -18.3 | to | 15.8 | ) |  | |  |
|  |  | Semimembranosus | 24.9 | ± | 10.7 | -4.0 | ± | 10.8 | 22.4 | ± | 9.0 | -4.5 | ± | 10.8 | 0.5 | | ( | -9.7 | to | 10.7 | ) |  | |  |
|  |  |  |  |  |  |  |  |  |  |  |  |  |  |  |  | |  |  |  |  |  |  | |  |
| Relative | | Biceps femoris | 39.9 | ± | 6.1 | 0.4 | ± | 5.7 | 37.0 | ± | 10.0 | -1.8 | ± | 12.2 | 2.2 | | ( | -9.7 | to | 11.5 | ) | *0.597* | |  |
| contribution | | Semitendinosus | 29.7 | ± | 10.2 | -3.5 | ± | 8.0 | 34.3 | ± | 5.4 | 0.9 | ± | 8.4 | -4.4 | | ( | -12.2 | to | 3.4 | ) |  | |  |
| (%con) |  | Semimembranosus | 30.9 | ± | 7.6 | 3.1 | ± | 9.2 | 28.7 | ± | 8.2 | 0.9 | ± | 11.4 | 2.2 | | ( | -7.7 | to | 12.0 | ) |  | |  |
|  |  |  |  |  |  |  |  |  |  |  |  |  |  |  |  | |  |  |  |  |  |  | |  |

*Values are means ± standard deviations, Δ; follow-up minus baseline, CI; confidence interval, p; probability value interaction effect, %MVIC; percentage maximal voluntary contraction, %con; percentage relative contribution.*

**
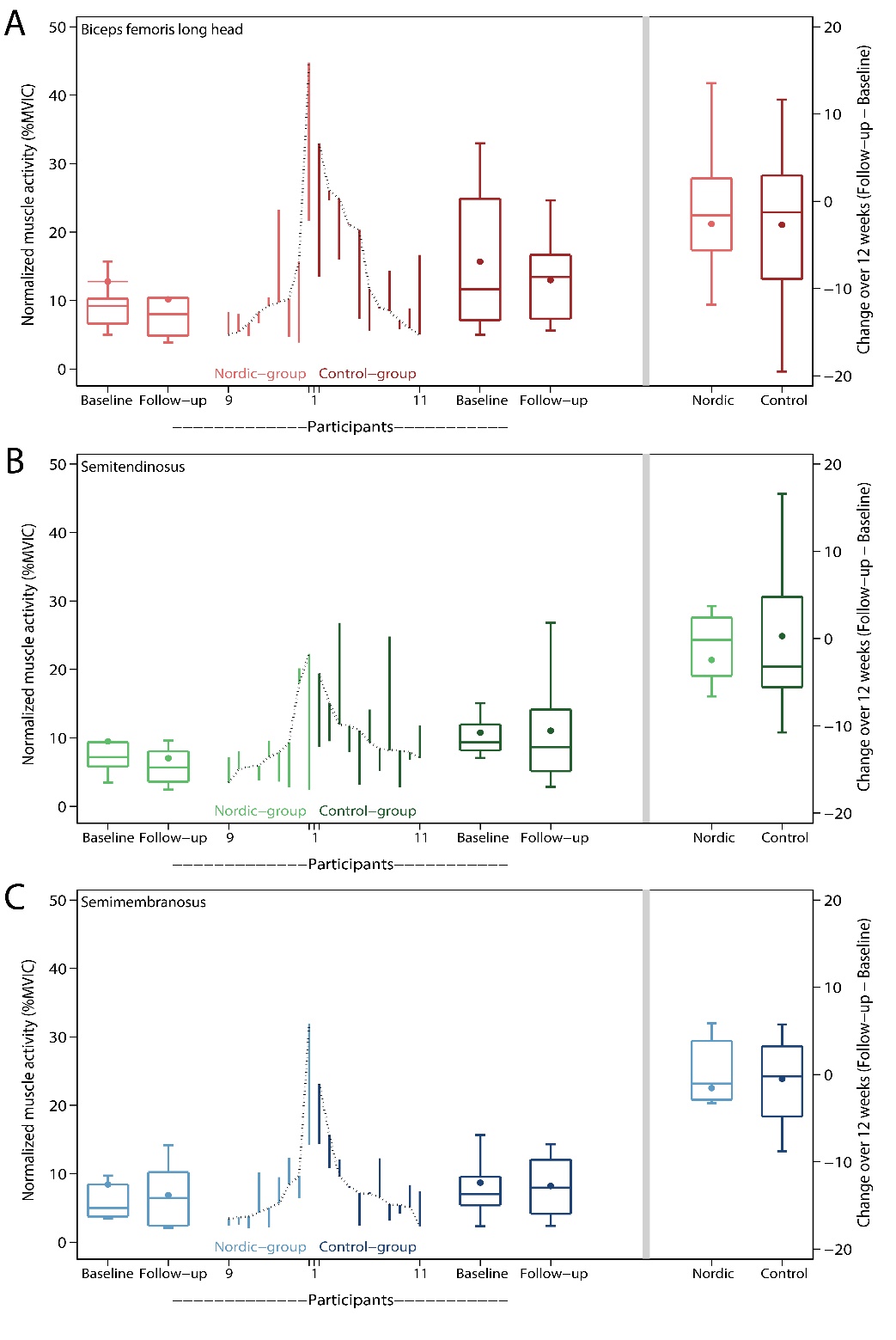
**

**Figure S3.** Change in normalized muscle activity for each participant of the Nordic- and Control-group over 12 weeks. Muscle activity was measured through multichannel electromyography in the early-swing phase of high-speed running. (A) biceps femoris long head. (B) semitendinosus. (C) semimembranosus. Changes from baseline to 12 weeks are represented by the vertical lines. Upward and downward lines indicate increases or decreases, respectively. The horizontal lines in the boxplots from bottom to top represent the 25^th^, 50^th^ (median) and 75^th^ percentile. The dots within the boxplot represent the mean. The whiskers show the lowest and highest value.

**
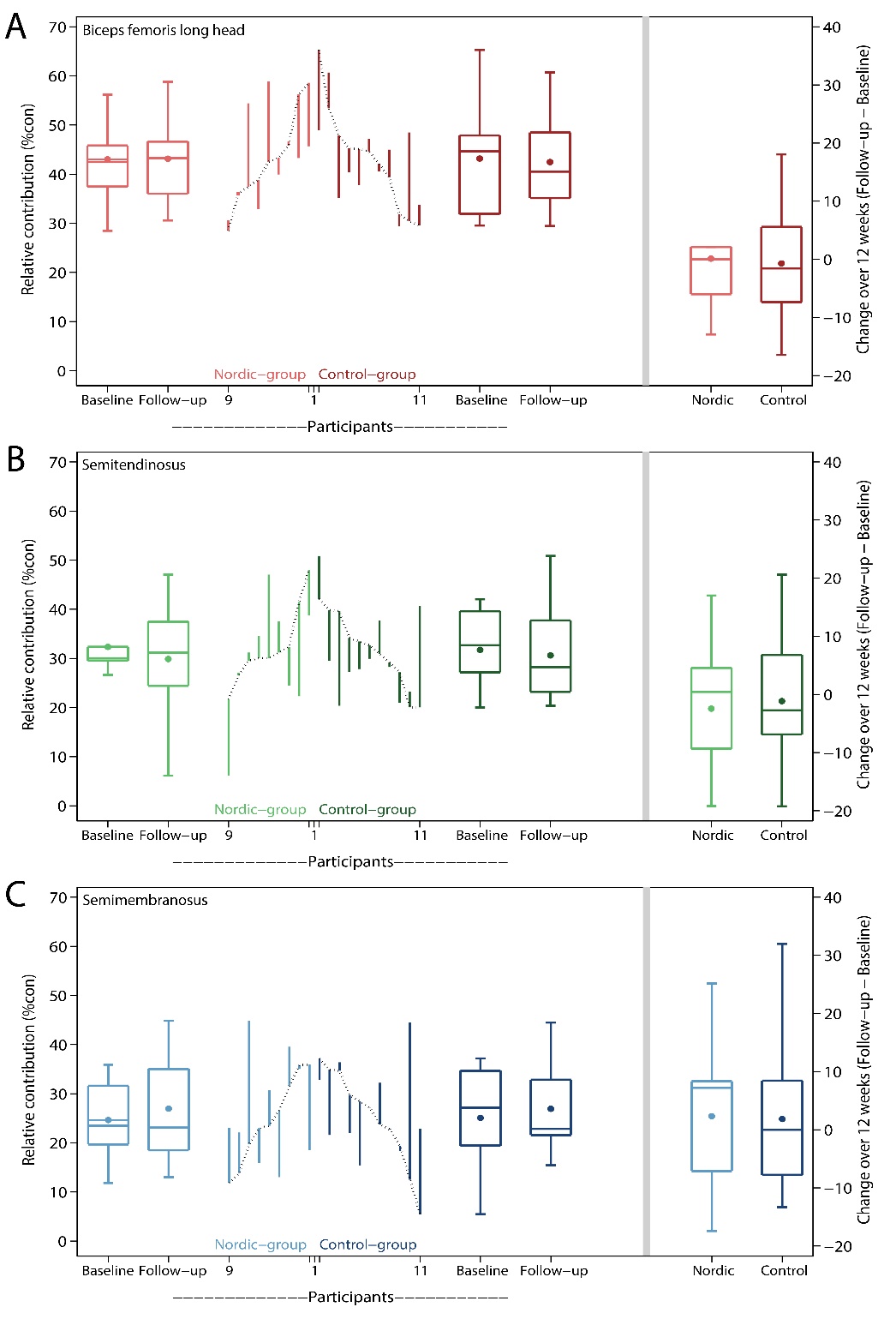
**

**Figure S4.** Change in relative contribution for each participant of the Nordic- and control-group over 12 weeks. Contributions were measured through multichannel electromyography in early-swing phase of high-speed running. (A) biceps femoris long head. (B) semitendinosus. (C) semimembranosus. Changes from baseline to 12 weeks are represented by the vertical lines. Upward and downward lines indicate increases or decreases, respectively. The horizontal lines in the boxplots from bottom to top represent the 25^th^, 50^th^ (median) and 75^th^ percentile. The dots within the boxplot represent the mean. The whiskers show the lowest and highest value.

**
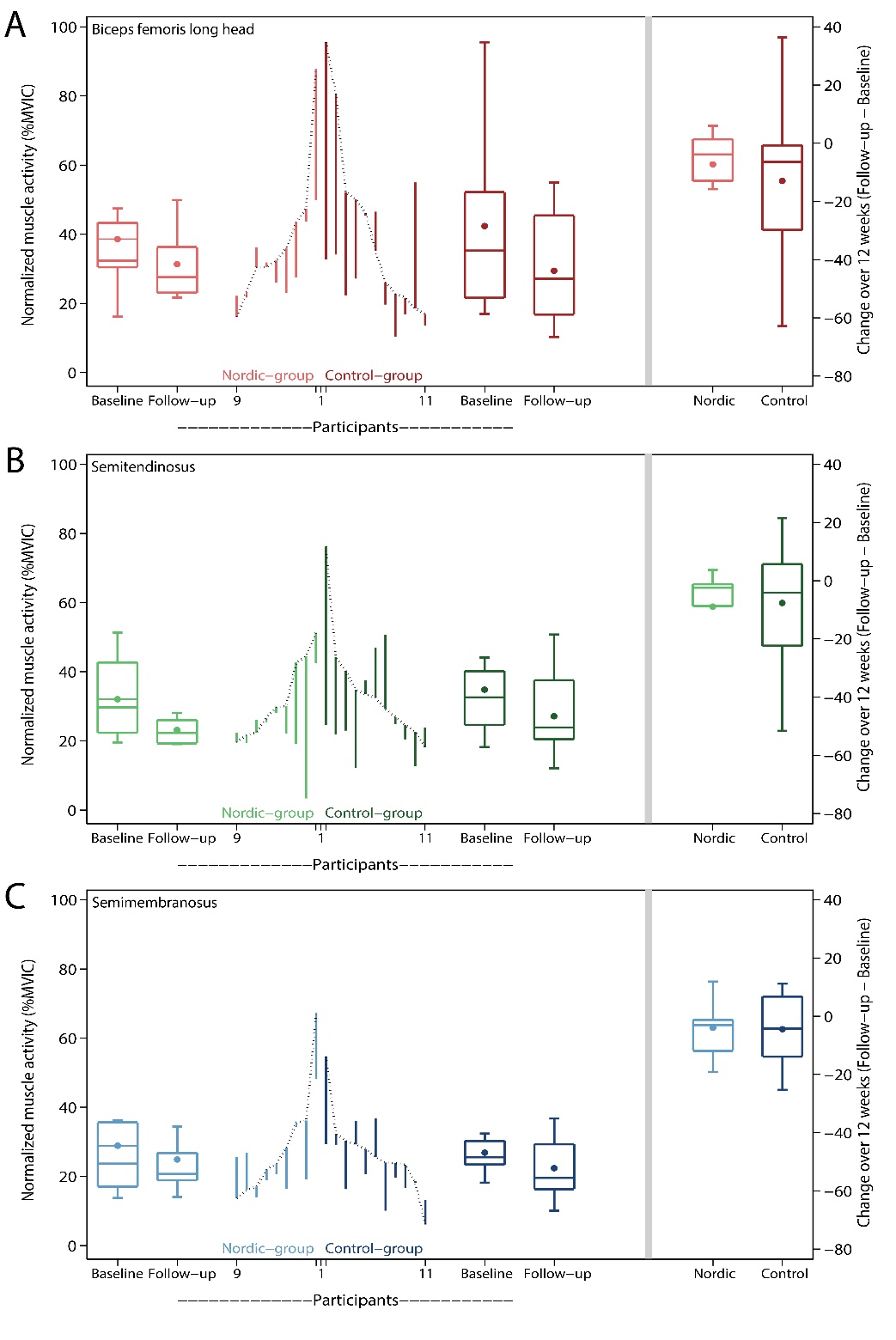
**

**Figure S5.** Change in normalized muscle activity for each participant of the Nordic- and Control-group over 12 weeks. Muscle activity was measured through multichannel electromyography in the stance phase of high-speed running. (A) biceps femoris long head. (B) semitendinosus. (C) semimembranosus. Changes from baseline to 12 weeks are represented by the vertical lines. Upward and downward lines indicate increases or decreases, respectively. The horizontal lines in the boxplots from bottom to top represent the 25^th^, 50^th^ (median) and 75^th^ percentile. The dots within the boxplot represent the mean. The whiskers show the lowest and highest value.

**
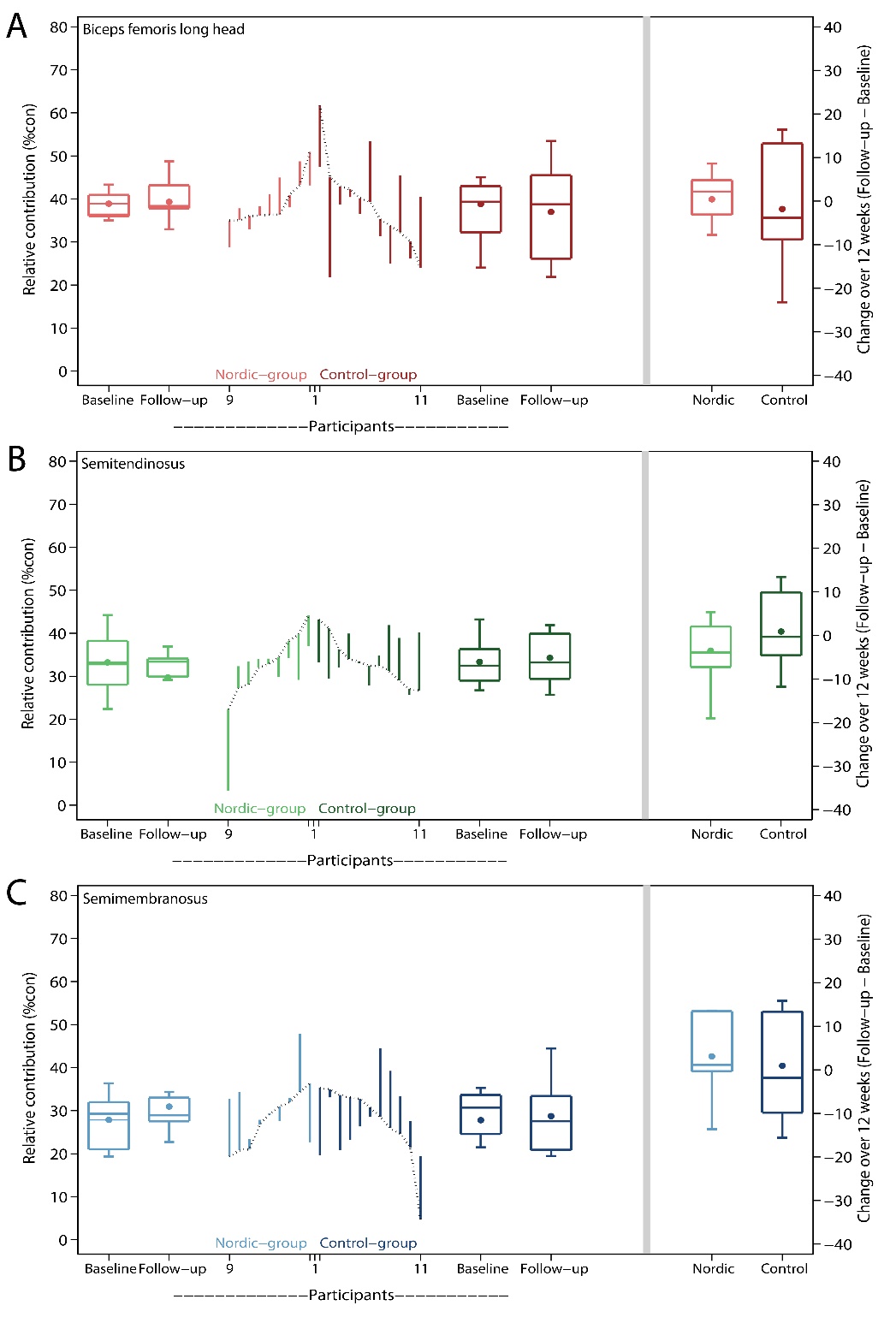
**

**Figure S6.** Change in relative contribution for each participant of the Nordic- and control-group over 12 weeks. Contributions were measured through multichannel electromyography in stance phase of high-speed running. (A) biceps femoris long head. (B) semitendinosus. (C) semimembranosus. Changes from baseline to 12 weeks are represented by the vertical lines. Upward and downward lines indicate increases or decreases, respectively. The horizontal lines in the boxplots from bottom to top represent the 25^th^, 50^th^ (median) and 75^th^ percentile. The dots within the boxplot represent the mean. The whiskers show the lowest and highest value.
